# Neuromusculoskeletal Arm Prostheses: Personal and Social Implications of Living With an Intimately Integrated Bionic Arm

**DOI:** 10.3389/fnbot.2020.00039

**Published:** 2020-07-24

**Authors:** Alexandra Middleton, Max Ortiz-Catalan

**Affiliations:** ^1^Department of Anthropology, Princeton University, Princeton, NJ, United States; ^2^Center for Bionics and Pain Research, Mölndal, Sweden; ^3^Department of Electrical Engineering, Chalmers University of Technology, Gothenburg, Sweden; ^4^Operational Area 3, Sahlgrenska University Hospital, Mölndal, Sweden; ^5^Department of Orthopaedics, Institute of Clinical Sciences, Sahlgrenska Academy, University of Gothenburg, Gothenburg, Sweden

**Keywords:** prosthetics, implanted electrodes, qualitative research, social studies of science and technology, human–machine interface

## Abstract

People with limb loss are for the first time living chronically and uninterruptedly with intimately integrated neuromusculoskeletal prostheses. This new generation of artificial limbs are fixated to the skeleton and operated by bidirectionally transferred neural information. This unprecedented level of human–machine integration is bound to have profound psychosocial effects on the individuals living with these prostheses. Here, we examined the psychosociological impact on people as they integrate neuromusculoskeletal prostheses into their bodies and lives. Three people with transhumeral amputations participated in this study, all of whom had been living with neuromusculoskeletal prostheses in their daily lives between 2 and 6 years at the time of the interview. Direct neural sensory feedback had been enabled for 6 months to 2 years. Participants were interviewed about their experiences living with the neuromusculoskeletal prostheses in their home and professional daily lives. We analyzed these interviews to elucidate themes using an interpretive phenomenological approach that regards participants’ own experiences as forms of expertise and knowledge-making. Our participant-generated results indicate that people adapted and integrated the technology into functional and social arenas of daily living, with positive psychosocial effects on self-esteem, self-image, and social relations intimately linked to improved trust of the prostheses. Participants expressed enhanced prosthetic function, increased and more diverse prosthesis use in tasks of daily living, and improved relationships between their prosthesis and phantom limb. Our interviews with patients also generated critiques of the language commonly used to describe human-prosthetic relations, including terms such as “embodiment,” and the need for specificity surrounding the term “natural” with regard to control versus sensory feedback. Experiences living with neuromusculoskeletal prostheses were complex and subject-dependent, and therefore future research should consider human–machine interaction as a relationship that is constantly enacted, negotiated, and deeply contextualized.

## Introduction

**FIGURE 1:**
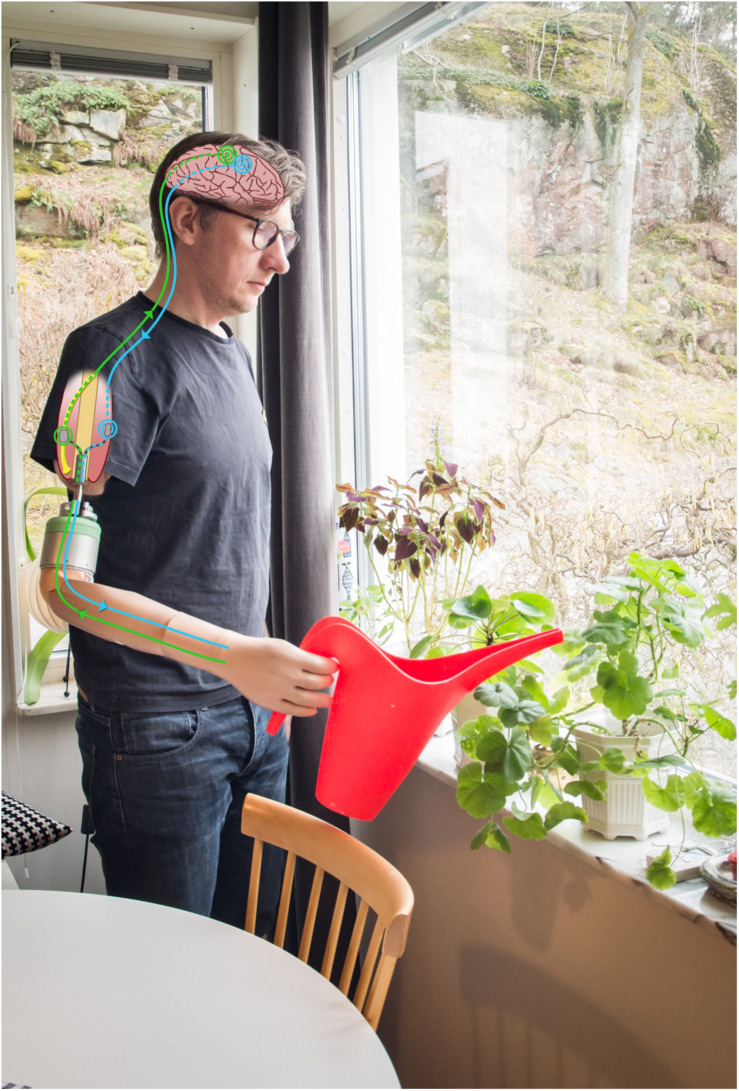
A neuromusculoskeletal arm prosthesis. An artist’s rendering of the signal chain of bidirectional communication between the prosthesis and neuromuscular system via implanted electrodes and a percutaneous osseointegrated implant system.

Prosthetic research and development have long sought to replace a lost biological limb with a functionally equivalent artificial one. In the early 1970s, researchers realized that implanted electrodes could provide superior control (Hoffer and Loeb, 1980; Stein et al., 1980), as well as intuitive sensory feedback via direct nerve stimulation (Clippinger et al., 1974, 1981). Recent work has provided further evidence on functional improvements enabled by implanted neuromuscular interfaces (Wendelken et al., 2017; Schiefer et al., 2018; Valle et al., 2018; Mastinu et al., 2019, 2020; Zollo et al., 2019). However, clinical implementation of these efforts had been hindered by the lack of a safe and long-term stable bidirectional interface between implanted electrodes and external prosthetic limbs. Neuromusculoskeletal prostheses, a novel concept in artificial limb replacement, solves this longstanding problem by utilizing a percutaneous osseointegrated implant for direct skeletal attachment of the prosthesis to the body, while also providing bidirectional interfacing to the user’s neuromuscular system via implanted electrodes in nerves and muscles (Ortiz-Catalan et al., 2014, 2020) (Figure 1).

Three participants (P1, P2, and P3) with unilateral transhumeral amputations were implanted with neuromusculoskeletal limb prostheses and have used them in daily life for over 7 (P1) and 3 (P2 and P3) years without interruption (Ortiz-Catalan et al., 2020). Two participants (P2 and P3) also received targeted muscle reinnervation for intuitive control of their prosthetic hand (Kuiken et al., 2009). Whereas the long-term home use of non-invasive sensorimotor prosthetic systems has been studied with surface electrodes (Schofield et al., 2020), this was the first time people with limb loss could use implanted electrodes to control and receive somatosensory feedback from their prostheses in their daily lives unsupervised and outside the constraints of research laboratories. This breakthrough, which at first glance appears purely technological, has important social consequences, as humans once deprived of an extremity are now living with an intimately integrated artificial limb connected to their skeleton, nerves, and muscles. Quantitative investigations, while indicative of the technology’s stability and performance, tell only part of the story. They do not speak directly to the human side of the human–machine relationship. Here, we address for the first time the personal and social experiences of those living with such highly integrated bionic limbs used chronically and ecologically in their environments.

Whereas qualitative research has been limited and conducted in the context of less intimately integrated limb prostheses (Murray, 2004; Lundberg et al., 2011; Widehammar et al., 2018; Cuberovic et al., 2019; Franzke et al., 2019; Graczyk et al., 2019; Hansen et al., 2019), it has nevertheless shown that the perspectives and opinions of those impacted by such medical interventions form a particular kind of evidence and expertise (Murray, 2004). The embodied knowledge (Merleau-Ponty, 1962; Bourdieu, 1990) produced from firsthand experience is unique from data gathered from traditional quantitative methods, serving to complement and at times even challenge quantitative data. Science is a practice of both knowledge-making *and* meaning-making. In our particular case, this relates to how humans experience the world they inhabit and how they create meaning from said experiences. Tending to meaning-making as an integral part of knowledge-making is crucial when studying the human impact of embodied biomedical technologies and served as a motivation for this study. Incorporating qualitative perspectives of those directly impacted by biomedical interventions can offer a more holistic, nuanced understanding of these phenomena, with the capacity to influence both their development and practice (Long et al., 2006).

**FIGURE 2:**
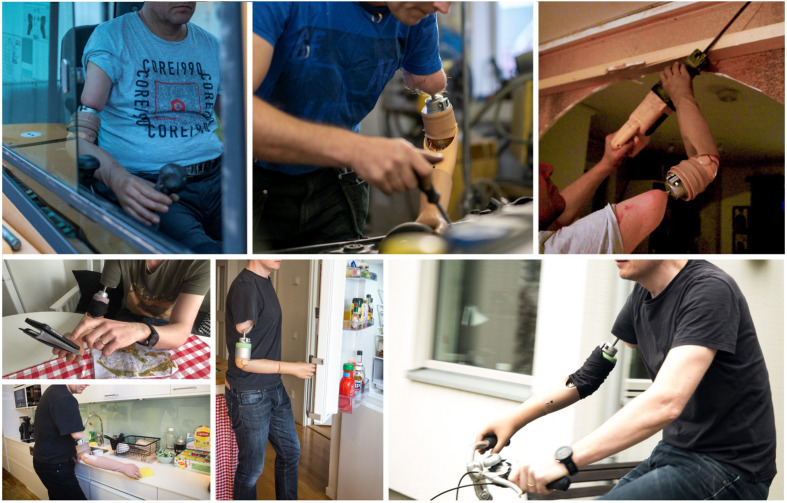
Neuromusculoskeletal prostheses used in daily life. Participants used an arm prosthesis directly interfaced to their skeleton, nerves, and muscles (neuromusculoskeletal) in professional and personal activities of the daily living for over 7 years. The prostheses do not require additional computational or powering equipment that is not already contained within the prosthetic arm itself (self-contained).

This study is motivated by patient-driven knowledge about the experience of living with neuromusculoskeletal limb prostheses in patients’ own homes and social worlds, outside the laboratory and clinical confines (Figure 2). To better understand how and to what extent people incorporate these artificial limbs into their lives and senses of body and self, we conducted in-depth, semi-structured interviews (Bernard, 2006) with the three aforementioned participants. We chose interviews as opposed to questionnaires because we wanted to understand the stories and experiences of patients through open-ended questions and explore more deeply the themes offered by patients *in situ*, unearthing greater detail from their stories than possible in questionnaire form. We used an interpretive phenomenological approach (IPA) for thematic content analysis (Holloway and Todres, 2003; Sandelowski and Barroso, 2003; Smith et al., 2012), which places peoples’ experiences and ways of knowing at the center, as lenses to understand lived phenomena. We chose IPA as our analytical tool because this method is best suited for approaching peoples’ lived experiences not as objective realities passively perceived (Brocki and Wearden, 2006), but rather actively crafted through peoples’ own processes of interpretation and sense-making. IPA was more appropriate to this end than discourse analysis (DA). DA largely bypasses subjects’ cognition and perception, focusing instead more linearly on the relationship between respondents’ verbal statements and pre-existing discourses (Smith et al., 2012), of which there are few in this emerging phenomenon of neuroprosthetics, particularly from first-person patient perspectives. IPA is also more suitable than grounded theory methodology (Creswell, 2007) because with a sample size of three patients, we did not seek to produce a model universalizing patient experience, but rather to attend to the particularities inherent in this very nascent and emerging human–machine interface. While these first accounts of living with a neuromusculoskeletal prosthesis can help illuminate how users relate to and make sense of intimately integrated biomedical technologies, a more robust sample size and longitudinal study would be needed to derive any grounded theory of significance.

In concert with IPA, we analyzed themes generated by the data itself as opposed to preordained categories. While we prioritized the themes common among all three participants, we also create space for nuances (i.e., noting when one patient raised a perspective not articulated by the other two). Although IPA encourages “dropping” themes not robustly provided in the data, the particularity or singularity of these findings do not necessarily indicate their insignificance. Rather, they indicate that people’s values and experiences regarding a phenomenon are inherently nuanced and varied. As the first author is an anthropologist, paying attention to such differences remains important, spawning further research inquiry. What ensues is a depiction of said nuances as well as more generalizable themes as they relate to peoples’ firsthand experiences living with neuromusculoskeletal prostheses.

## Materials and Methods

### Experimental Design

An interpretive phenomenological approach (IPA) for thematic content analysis (Holloway and Todres, 2003; Sandelowski and Barroso, 2003; Smith et al., 2012) of semi-structured deep interviews was employed to understand the lived phenomena of uninterrupted, unmonitored home use of the neuromusculoskeletal limb prostheses. At the time of the interviews in February 2019, subjects had been using the system for a period of 6 (P1) or 2 (P2 and P3) years. Participants have continued to use the system up to the publication of this manuscript. In order to reduce biases due to patient compliance, the interviewer was independent from the healthcare and technology providers.

### Neuromusculoskeletal Arm Prostheses

The neuromusculoskeletal prosthesis consists of a percutaneous osseointegrated implant (skeletal interface), implanted electrodes in nerves and muscles (neuromuscular interfaces), and signal transfer mechanisms embedded in the skeletal interface enabled by bidirectional communication between the external prosthesis and the internal neuromuscular interfaces. The osseointegrated implant was based on the OPRA implant system (Integrum AB, Sweden) originally used for transfemoral amputations (Brånemark et al., 2001, 2019) and later employed in upper limb amputations (Jönsson et al., 2011). This implant system was further developed to include signal feed through mechanisms and implanted neuromuscular electrodes (e-OPRA), effectively serving as a neuromusculoskeletal human-machine interface (Ortiz-Catalan et al., 2014, 2020). Epimysial electrodes were implanted on remnant muscles as a source for prosthetic control, and spiral cuff electrodes were wrapped around severed nerves to deliver electrical stimulation for sensory feedback. P2 and P3 received targeted muscle reinnervation (TMR) surgery (Kuiken et al., 2009), in which the ulnar nerve was transferred to the short head of the biceps muscle and the distal branch of the radial nerve was transferred to the lateral head of the triceps muscle (Ortiz-Catalan et al., 2020). Myoelectric signals from the reinnervated muscles were observed as soon as 4 weeks after surgery (Mastinu et al., 2018). A custom-designed embedded system within the prosthetic arm was used to control the prosthesis using signals from the epimysial electrodes and to deliver electrical stimulation via the cuff electrodes (Mastinu et al., 2017). The use of the percutaneous osseointegrated implant as a means of bidirectional communication between the prosthesis and the implanted electrodes, as opposed to mechanically unstable percutaneous leads, allowed for the long-term and uninterrupted use of the prosthetic system in daily life (Ortiz-Catalan et al., 2020).

### Participants

Three people, all Swedish males with upper-limb transhumeral amputations, participated in this study. Since provided with the neuromusculoskeletal prostheses 4–6 weeks after implantation, all subjects have worn them all the time while awake, except while showering or swimming. No special training or rehabilitation was provided for the subjects to start utilizing their neuromusculoskeletal prostheses, as these subjects had used myoelectric prostheses in the past. Participants were not paid to participate in this study other than reimbursement of their travel costs. Their backgrounds are described subsequently.

#### Participant 1

Participant 1 (P1), a 46-year-old male, had his right arm amputated due to a malignant tumor in 2003. He used a conventional myoelectric prosthesis with two surface electrodes and socket suspension until 2009, when he was operated with a percutaneous osseointegrated implant for bone-anchoring of the prosthesis. He used a myoelectric prosthesis with surface electrodes and direct skeletal attachment until he became the first subject to be implanted with the neuromusculoskeletal prosthesis in 2013 (Ortiz-Catalan et al., 2014). Between 2013 and 2017 he only used the implanted electrodes for prosthetic control without sensory feedback. Since 2017, he has used the implanted electrodes for control and sensory feedback in daily life. He has commanded the prosthetic hand (SensorHand, Ottobock, Germany) using two electrodes via “direct control” (one myoelectric signal activates one action in the prosthesis) and locking/unlocking the elbow using co-contraction (ErgoArm, Ottobock, Germany). At the time of the interview, P1 had been living with the new neuromusculoskeletal prosthesis at home for the past 6 years (2 years with closed-loop control), sometimes even sleeping while wearing it. He works as a truck driver and deliverer, with a physical job that demands carrying heavy loads. He lives with his partner and three children, and enjoys skiing, ice fishing, and snow scootering in his free time.

#### Participant 2

Participant 2 (P2), a 45-year-old male, lost his left arm in a high-voltage electrocution accident while working as an electrician in 2011. He underwent osseointegration surgeries in 2014 (Jönsson et al., 2011). From 2014 to 2017 he lived with an osseointegrated prosthetic and two surface electrodes. In January 2017, P2 received implanted electrodes as part of the neuromusculoskeletal interface. He used the implanted electrodes without sensory feedback until 2018 when the sensory feedback was enabled to be used in daily life. He commanded the prosthetic hand (SensorHand, Ottobock, Germany) using “direct control” from two native muscles until 10 weeks after surgery, when the control was switched to the two TMR muscles (Mastinu et al., 2018). He locks/unlocks the elbow using co-contraction (ErgoArm, Ottobock, Germany). At the time of the interview, he had been using the neuromusculoskeletal prosthesis in daily life for 2 years (6 months with closed-loop control). He currently works as a project leader for an installation company, where he heads the electricity division. P2 lives with his wife and three children, and enjoys rally racing and working on cars in his spare time.

#### Participant 3

Participant 3 (P3), a 43-year-old male, lost his right arm in a work accident as a seaman at sea in 1997, at the age of 22. As he puts it, “I’ve lived half my life with two arms and half my life with one arm.” P3 first received a socket prosthesis in 1997, the summer after his amputation. After 5 years of use, he abandoned the prosthesis due to its cumbersome nature, preferring to live without one for nearly 12 years. During this time, he developed concerns that his body was becoming “crooked” due to the compensation and overuse of one side. He also started developing back pain and spasms. In 2014 he was operated with osseointegration and began using a myoelectric prosthesis again with two surface electrodes. In January 2017, he was implanted with the neuromusculoskeletal interface. He used the implanted electrodes for control without sensory feedback until 2018, when sensory feedback was enabled for closed-loop control in daily life. He commanded the prosthetic hand (SensorHand, Ottobock, Germany) using “direct control” from two native muscles until 40 weeks after surgery, when the control was switched to the two TMR muscles (Mastinu et al., 2018). He locks/unlocks the elbow using co-contraction (ErgoArm, Ottobock, Germany). At the time of the interview, he had been using the neuromusculoskeletal prosthesis at home in daily life for 2 years (6 months with closed-loop control). P3 is an IT consultant, an athletic individual who enjoys orienteering, running, canoeing, and skiing. He lives with his wife and two children.

### Data Collection

The first author, who is independent from the development team and a medical anthropologist conducting a larger ethnographic study about patient experiences living with neuromusculoskeletal prosthetics, conducted in-depth, semi-structured interviews (Bernard, 2006) with each of the participants, ranging from 40 to 75 min. An interview guide can be found in the Supplementary Material (S1) based loosely on the work by Hansen et al. (2019). A framework of questions was used for each interview, beginning with more general questions to establish rapport and learn about the participant’s life, then focusing upon the themes of the participant’s history with prosthetics, prosthetic function and control, use in various home and daily life settings and environments, experiences of sensory feedback, and experiences with the phantom limb (pain and sensation). These questions were used to structure the conversation, but the interviewee led the way, making free associations and asked by the interviewer to expand and comment upon them. These interviews were conducted in the participants’ native language, Swedish, and audio recorded. Audio files of the interviews were then transcribed into Swedish by a professional transcription service and then translated into English by the first author.

### Data Analysis

This study aimed to place the firsthand experiences and perspectives of participants living with neuromusculoskeletal prostheses at the center, focusing on how people make meaning from said experiences to incorporate a device into their lives and sense of body and identity. From these experiences and firsthand reports, we sought to elucidate themes that spoke to the unique knowledge and expertise generated by prosthesis users themselves. Interviews were recorded in the participants’ native language (Swedish), transcribed, and then translated to English for thematic coding using the software NVivo (QSR International, Australia) in preparation for further analysis.

The first author read interview transcripts, identifying repeating patterns, categories, and themes present. The first author then cross-validated these themes with the second author. From the agreed-upon themes, the first and second authors iteratively derived a descriptive coding system, with several umbrella categories containing subthemes. To organize data according to these codes, the software NVivo 12 was used (NVivo qualitative data analysis Software; QSR International Pty Ltd. Version 12, 2018). NVivo is a tool for organizing sections of text according to codes (called “nodes”) generated by the user. See Table 1 for the code categories, themes, subthemes, and descriptions used.

**TABLE 1:** NVivo coding structure of categories and themes derived from participant interviews.

**Category/node**	**Sub-themes**	**Participants’ descriptions of:**
Mechanical attachment of prosthesis to body	Past experiences with socket prosthesis	Past experiences and practices using and wearing a socket prosthesis.
	Comparisons between socket and osseointegration	Comparison between participants’ past experiences with socket and current with osseointegration.
	Bodily adjustments and accommodations to prosthesis	Posture, pain in other parts of the body, compensation, numbness and tingling in other body parts (not missing body part), or lack thereof, for both socket and osseointegrated prostheses.
	Removing and putting on the prosthesis	Experiences with removal and attachment of the device, for both socket and osseointegration.

Control of prosthesis by user	Surface electrode experiences	Past experiences wearing and using surface electrodes, putting them on, challenges faced.
	Implanted electrode experiences	Current experiences with implanted electrodes.
	Electrical interference	Experiences with electrical interference from environment with prosthesis’s electrical system.
	Trust in the prosthesis	Participants’ degree of trust in prosthesis to not malfunction.
	“Naturalness” of control of prosthesis	The degree to which intuitive control of the prosthesis feels “natural.”
	Scenarios of use facilitated by control	New scenarios and occasions in which use is facilitated by improved control.
	Habituation and training	The training required to habituate body and prosthesis.
	Breakdown and malfunction	Challenges with control, breakdown and malfunction of the device.
	Description of feedback’s sensory qualities	Language about the quality or type of sensation users experience with regard to touch, location, size/area, frequency, and duration.

Experience of sensory feedback via neurostimulation	Sensory discrimination	Location of sensor contact with object and prosthetic hand in relation to felt sensation in the phantom hand.
[-10pt]	Appraisal of sensory feedback’s utility	Opinions regarding the utility, purpose, relevance, or quality of sensory feedback.
	Reliance on other forms of feedback	Other non-sensory (i.e., visual and auditory) feedback used to locate prosthesis in space or exercise control.
	The term “natural” with regards to sensory feedback	Invocation and use of the word “natural” to describe (or purposely not describe) different elements of sensory feedback.
	Stump sensation	Presence or absence of sensation or pain on the stump or residual limb.

Prosthesis use in daily life	Extent of usage	Amount of time prosthesis is used, including periodic removal and reattachment throughout the day, charging requirements.
	Diversity of tasks and activities of use	The tasks and activities participants use prosthesis for, comparison with past socket prosthesis and/or surface electrode activities of use.

Relationship between prosthesis and phantom limb	Phantom limb pain	The presence or absence or degree of phantom limb pain with and without prosthesis on, before and after use, and general patient history of phantom limb pain.
	Phantom limb position	The position of the phantom limb with and without the prosthesis.
	Phantom limb mobility	The mobility of the phantom limb with and without the prosthesis.
	Phantom limb sensation	Phantom limb sensation, particularly with respect to its relationship with neurostimulation for sensory feedback.

Self-esteem, self-image, and incorporation of prosthesis into body	Self-efficacy and independence	Participants’ sense of being independent and self-efficacious with regards to performing tasks and activities themselves.
	Self-esteem	Participants’ self-esteem before and after neuromusculoskeletal prosthesis, including comments on self-image, body-image, and identity.
	Feeling “handicapped”	The term “handicapped” and explanations of its meaning, its relevance to prosthesis use and function, as well as overall self-image in a societal context.
	Mood	Mood state and overall affective wellbeing before and after receiving a neuromusculoskeletal prosthesis.
	Ownership and prosthesis as “part of me”	The degree to which participants consider prosthesis part of their body, self, and/or identity.
	Prosthesis as tool	The degree to which participants experience prosthesis as an external tool.

Social and emotional wellbeing	Relationships with family members	Family members’ perceptions of neuromusculoskeletal prosthesis, interactions with family members in relation to neuromusculoskeletal prosthesis.
	Relationships with friends and coworkers	Friends’ and coworkers’ perceptions of neuromusculoskeletal prosthesis, interactions with friends and coworkers in relation to neuromusculoskeletal prosthesis.
	Interactions in public with strangers	Interactions with strangers in public with regard to the neuromusculoskeletal prosthesis.

From the NVivo coding, we interpreted themes and subthemes using interpretive phenomenological analysis (IPA) (Holloway and Todres, 2003; Sandelowski and Barroso, 2003; Smith et al., 2012). This entailed suspending our own expectations about the data and instead focusing on how participants articulated making a sense of meaning from their experiences. It is important to note that these themes and the coding system were generated from the data, as opposed to predetermined prior to the interview. IPA is derived from the philosophical and theoretical contributions of Martin Heidegger, whose phenomenology centers upon the embeddedness of the human subject in the world as “being in the world,” and thus focuses on the emic perspective of subjects themselves (Heidegger, 1927; Horrigan-Kelly et al., 2016). The method espoused in this study acknowledges that the researchers are also subjects, making sense of participants’ narratives (Smith and Osborn, 2015); thus, interpretation is an intersubjective process.

## Results

**FIGURE 3:**
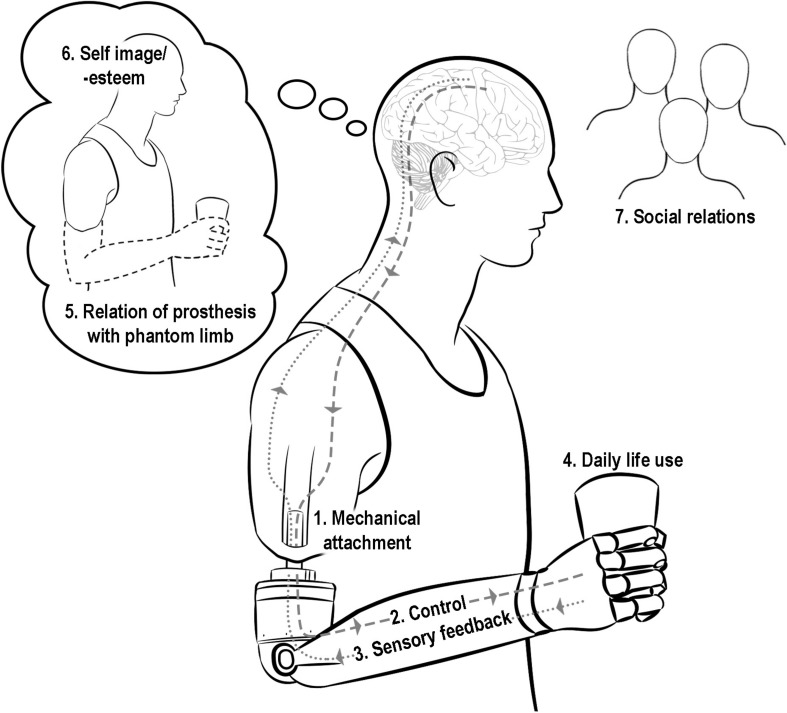
Diagram of the seven key themes derived from participant interviews. The seven themes include three exogenous elements (introduced by the intervention): (1) mechanical attachment of the prosthesis to the body, (2) intuitive control of prosthesis, and (3) the experience of sensory feedback; and four endogenous elements (resulting from patients’ experiences with said intervention): (4) practices and use of the prosthesis in daily life, (5) relationship of the prosthesis with the phantom limb, (6) self-image and self-esteem, and (7) social relations.

Our thematic content analysis of the interviews yielded themes largely grouped into seven categories. Three categories were exogenous elements introduced by the intervention: (1) mechanical attachment of the prosthesis to the body, (2) intuitive control of the prosthesis, and (3) the experience of sensory feedback. Four categories were endogenous elements resulting from patients’ experiences with said intervention: (4) practices and use of the prosthesis in daily life, (5) relationship of the prosthesis with the phantom limb, (6) self-image and self-esteem, and (7) social relations (Figure 3).

### Category 1: Mechanical Attachment

#### Participants Preferred Direct Skeletal Attachment via Osseointegration Over Socket Suspension of Prosthesis

All participants, unsolicited, invoked comparison between their past experiences with socket suspension as the means of attaching their prosthesis to their own body, and their current osseointegrated prosthesis with direct skeletal fixation. Participants used the words “uncomfortable,” “sweaty,” and “impractical” to describe their prior socket prostheses, in contrast to the words “comfortable,” “easy,” and “pleasant” to characterize their osseointegrated prostheses. P2 described deleterious effects in other parts of his body as a result of compensating to adapt to the socket fitting and overusing his intact arm: “I started getting crooked in the back and I lost sensation in the (remaining) hand’s fingers, and I thought ‘this won’t work long-term.” Furthermore, P2 described feeling the stump moving around independently and asynchronously inside the socket when attempting to perform movements. P1 and P3 also reported greater degrees of mobility and decreased associated bodily discomfort and pain when they switched from a socket to direct skeletal attachment.

All patients reported using their neuromusculoskeletal prostheses for longer periods than they did their socket prostheses. “It’s pleasant. I don’t get tired of having it on me,” P1 reflected. P3 was the most minimal user of his prior socket prosthesis (“It just hung there…As soon as I got home, I took off my prosthesis”), eventually abandoning his socket prosthesis for 12 years prior to osseointegration. Today, all patients use their prostheses for all waking hours of the day.

Patients also reported feeling more incentivized to remove and put on the neuromusculoskeletal prosthesis in occasions where they previously would not. P2 described this shift in use:

“Say (before bed) I’ve … showered … and taken off the prosthesis … Had it been a socket prosthesis, I would have never put it back on again, because it’s such a mess to get it to sit right. Then to set the electrodes (on the skin), find and get them to work, it’s not worth it. But (with osseointegration) one can just click (the prosthetic arm) into place and then it works again. One might think that’s a small thing, but that is quality of life.”

### Category 2: Control of the Prosthesis

#### Participants Experienced Improved Prosthetic Control With Implanted Electrodes

All three participants, unsolicited, drew comparison between surface electrodes and implanted electrodes, specifically emphasizing the increased sense of control gained from implanted electrodes. Recalling his time using surface electrodes, P2 recounted, “There were many disturbances. If I walked by an electromagnetic field or something (like a stove), I dropped things, or the elbow would activate…” Similarly, P3 remarked, “There are so many outer factors that can disturb (a myoelectric prosthesis with surface electrodes). It can open and close itself.”

All participants recalled instances of erratic hand movements with surface electrodes, prompted by electromagnetic interference in the environment. With implanted electrodes, all participants reported a greater degree of agency over the prostheses’ movements. As P2 described:

“All such disturbances are gone now with the implanted electrodes. It’s a lot smoother. I’m in better control. With implanted electrodes, it’s *me who decides* when I will open and close the hand.”

P2 recalled functional limitations in daily life activities with surface electrodes. “Eating with a knife and a fork using a socket prosthesis, that was … worthless.” These functional challenges were surmounted, P2 reported, once he changed from surface to implanted electrodes: “now I can hold a knife in my left hand and cut with it and make very small movements, and the prosthesis stays in place.”

#### Improved Control and Decreased Interference Strengthened Participants’ Trust of the Prosthesis, Engendering Prosthetic Use in More Diverse Scenarios

All participants said that interferences and disturbances with surface electrodes gave the sense that it was not them who was controlling the prosthesis, but rather other environmental factors. Consequently, participants expressed mistrust of the prosthesis with surface electrodes: “I couldn’t trust that I could carry something,” P2 explained. P3 also described the surface electrodes as “not really trustworthy.”

Participants drew a causal relationship between improved control and trust. As P3 described:

“It’s me who has control. I trust the prosthesis. I can carry a wineglass (now). A wineglass with wine in it. I would have never done that with a (surface electrode) prosthesis.”

In adapting to the increased functionality and control afforded by the implanted electrodes, participants described adjusting their tolerance levels of prosthetic function and malfunction, with higher expectations for their new neuromusculoskeletal system. For example, with the neuromusculoskeletal system, if P3’s hand did happen to open or close when he did not intend, this signaled to him “a malfunction in the hand or software.” P3 then sought to correct this malfunction with the engineering team, whereas with surface electrodes he would have “just accepted (that) as part of the limitations of the electrodes.”

#### Participants Described Functional Control of the Neuromusculoskeletal Prosthesis as Intuitive and “Natural”

All participants used the word “natural” to describe the “thought-steered” (as referred to by P2) control and responsiveness of their prostheses. P2 said that the benefits of the prosthesis are most apparent when he *isn’t* wearing it:

“When I don’t have the prosthesis on, I do so many small things that I don’t think about, because it’s become so natural (for me) to wear the prosthesis. I very rarely go without the prosthesis, but say I’ve taken it off if it’s run out of power or something, then I do things in the air because the prosthesis is gone. It has become so natural.”

### Category 3: The Experience of Sensory Feedback

At the time of the interviews, participants had been using neural sensory feedback in daily life for 2 years (P1) or 6 months (P2 and P3). Participants were provided with a conservative but biologically inspired neuromodulation strategy that consisted of modulating the frequency of stimulation proportional to grip force (5–30 Hz roughly corresponding to 5N to 25N) (Ortiz-Catalan et al., 2020). The maximum frequency of stimulation (30 Hz) was orders of magnitudes lower than the natural frequencies at which peripheral nerves can communicate information, but it had to be constrained owing to safety considerations (Günter et al., 2019). As expected, frequency discrimination was initially poor due to the limited bandwidth and required a stimulation frequency change of approximately 50% to be noticeable. Over time, a smaller difference of frequency of about 30% was required for the participants to perceive a change of intensity (Ortiz-Catalan et al., 2020). From the time of contact with an object, participants received sensory stimulation for 5 seconds, at which point stimulation was stopped for safety purposes (Günter et al., 2019).

#### Participants Did Not Describe Sensory Feedback as “Natural” and Expressed Doubt as to Whether It Needed to Be

All three participants used the words “electric” and “numb” to explain how the sensory feedback felt. P1 described the area of sensation as small, “like the point of a pen,” which then “grows outward” as the sensation increases. P3 recalls the first time he received sensory feedback:

“In the beginning it was very strange, to just, feel…I feel all of a sudden something I haven’t felt for so many years. I have not had any sensation in that way.”

When asked whether they considered the sensory feedback “natural-feeling,” all participants hesitated to describe it as such. P2 and P3 clarified that a “natural” sensation depends not only upon its sensory quality, but also its perceived location with respect to the sensor in the prosthetic hand. All participants used the same prosthetic hand (SensorHand, Ottobock, Germany) with the sensors located in the prosthetic thumb (center of the distal phalanx), and thus participants must press the thumb against an object to generate sensory feedback. However, owing to the placement of the neural electrodes and consequent lack of selectivity, the participants experienced the elicited sensation in various locations on their phantom hand other than the thumb (Ortiz-Catalan et al., 2020). P2 described himself as “lucky” because “the sensation (I feel) exists in the thumb…and the sensor (on the prosthetic hand) is located there, too, so it is quite natural…that (both) get to be in almost the same spot.” Yet P3 experienced the sensation elsewhere. “When I touch this,” he demonstrated during the interview, touching a water bottle with his thumb, “I have sensation there,” he pointed to the lateral side of his middle finger. According to P3, the discrepancy between the location of the sensor in the prosthetic hand (thumb) and location of the perceived sensations (middle finger) made the sensation feel “not natural.” When asked what a natural sensation would feel like, P3 said “we would feel where we touch.” P1, who experienced the sensory feedback in the palm, did not comment on the perceptual difference with regard to the sensor location on the hand.

When probed further, all participants expressed doubt whether “natural-feeling” was necessarily the most important goal of sensory feedback. Instead, they highlighted its functional benefits. P1 found it most important that the sensation merely exists, because it allows him to take grip of objects more confidently, often without relying on sight. P2 expressed skepticism that the sensation could ever feel “natural,” but made an important distinction that it need not feel natural in order to be helpful: “to find a sensation that feels natural, I think that’s very difficult, but to get a signal that is helpful, that can probably happen.”

#### Participants Attributed Limited Benefits to Current Sensory Feedback

Participants’ reports were not unanimous regarding the quality of their experiences of the sensory feedback. P1 described, “I feel two levels (of intensity). A lighter level when I grip lightly, and then when I grip a little harder, then it’s stronger.” In contrast to P1’s reported experience, P2 and P3 reported difficulty differentiating between intensity levels. Rigorous psychometric evaluations showed that an approximately 30% change in stimulation frequency was required for the participants to perceive a difference in intensity (Ortiz-Catalan et al., 2020); however, the available bandwidth for stimulation was limited (5–30 Hz), thus directly impacting the resolution of perception. Of all subjects, P1 voiced the most utility in knowing how hard he is gripping an object, reporting improved ability to handle delicate objects like his smartphone or a glass without breaking them. P2 described the sensation as “so weak that when I do something active, I don’t think it’s there.” As he explained:

“It works. It’s there. But I have not yet seen its benefit. I don’t really know what it’s going to be good for. When you do something with precision slowly and properly concentrate, then you feel the feeling, but the benefit…it’s difficult for me to see. The (sensory feedback) is there, but it doesn’t add anything for me.”

P3 expressed a similar reaction when asked about the utility of the sensory feedback:

“It’s exciting, interesting to see but I don’t know if it does so much more. There is of course big potential with sensory feedback, and everything must begin with something. But the way it is now, it’s mostly just exciting and cool.”

When prodded further to explore what he considered the biggest barrier or limitation of the current sensory feedback, P2 explained:

“To have a sensation that you’re grabbing something, that’s not so meaningful for me, because I see that I’m doing that. I would rather have a sensation where I feel like I’m *losing* the grip of something.”

#### Visual Feedback Remained Relied Upon to Supplement or Confirm Grip

Two participants (P2 and P3) reported needing to rely on visual feedback to supplement sensory feedback, while P1 reported that he can gauge a grip by feeling without having to look at the object. P2 explained using sight because “the sensation is not good enough” to rely on solely to grab an object out of sight. P2 referred to using sight as a supplemental sensation confirming his grasp. P3 compared the sensory feedback of his biological hand with his prosthetic:

“When I pick something up (with my biological hand) I feel it and I don’t need to look at it. But with the sensory feedback, to know ‘oh, I have taken that up,’ I must still have visual contact.”

#### Participants Reported Either No Change or Improvement in Stump Sensation

P1 and P3 reported no change in sensation in or around the residual limb and amputation site. P2 reported improvements with pain and sensation on the surface of residual limb. “Earlier it was quite sensitive,” he explained, “it could be tight and tingle…for a year after the operation (amputation) I was very sensitive with small shock (sensations).” This sensitivity, P2 reports, has gotten “much, much better.” None of the three participants could feel the electrodes inside their arm.

### Category 4: Practices and Use of Prosthesis in Daily Life

#### Participants Increased Amount of Time and Diversity of Daily Life Tasks Using the Neuromusculoskeletal Prosthesis

All participants reported an increase in the amount of time they wear the neuromusculoskeletal prosthesis during the day, compared to prior experiences with socket prostheses and/or surface electrodes. All reported wearing the neuromusculoskeletal prosthesis from waking up until going to sleep (“It’s among the first things I do: I put on my prosthesis, to the last thing I do: take it off” – P2) for periods ranging from 12 to 20 h. While P2 and P3 removed their prostheses overnight to charge its battery, P1 often slept with the prosthesis on, especially when traveling for work.

All participants also described performing an expanded diversity of tasks with the neuromusculoskeletal prosthesis, compared to a myoelectric socket prosthesis or osseointegration with surface electrodes. All participants emphasized increased involvement and participation in family chores and activities, including: cooking, washing the dishes, shoveling the snow, mowing the lawn, skiing, gardening, and hanging laundry. The only activities participants reported *not* using the prosthesis for were swimming, bathing, and running.

Each participant highlighted increased involvement in family life as the most beneficial element of increased prosthetic use, with P2 and P3 reporting feeling “more helpful” to their families. “I can do things faster than I could before,” P3 explained. P2 articulated that his needs for a prosthesis in everyday life are modest and simple, but that these very simple things matter most: “You don’t necessarily have to have a super advanced hand…it’s all about these small things.”

Participants also reported using their neuromusculoskeletal prostheses for work to varying degrees, depending on their professional demands. As a truck driver and deliverer, P1 explained that his work can be quite physical; he used the prosthesis not only for holding the wheel and steering while driving, but also tying anchoring chains for cargo and lifting heavy items off his truck. While P2 used his prosthesis for everyday office tasks, like collecting paper from the printer and stapling, he expressed more benefit at home than at work:

“Say the prosthesis breaks and I must go without it for a week (while it’s being repaired). I would suffer more at home than I do at work…I would miss it more.”

P2 attributed this difference to his work’s non-physical nature, compensated for by using his sound arm. Likewise, P3 works primarily on the computer, and he expressed minimal work-related functional benefits from his prosthesis. He described the prosthesis as “clumsy” when trying to type on a keyboard. Rather, P3 explained that the benefits he perceived at work with regard to his neuromusculoskeletal prosthesis were due to increased self-esteem, which in turn improved the quality of his work and relations with colleagues.

### Category 5: Relationship of the Prosthesis With the Phantom Limb

#### Participants Experienced Significant Decrease of Phantom Limb Pain

Two out of three participants (P1 and P3) reported having experienced phantom limb pain (PLP) prior to being implanted with the neuromusculoskeletal interface. P1 experienced PLP after his amputation and during the years he used a socket prosthesis. The pain diminished but still lingered after he received osseointegration, but “after they implanted the electrodes, it … disappeared.” Prior to osseointegration, P3 also experienced significant phantom limb pain, which presented as electrical shocks, or the feeling of something cutting into his hand. This pain made sleep difficult, waking him in the middle of the night and impacting his energy and quality of life. After receiving the neuromusculoskeletal interface, P1 and P3 reported that phantom limb pain ceased completely.

#### Participants Experienced Locational Synchrony Between Phantom Limb and Prosthesis Positions, as Well as Improved Mobility of Phantom Hand

All participants reported changes in the position and mobility of their phantom hand as a result of using the neuromusculoskeletal prosthesis. These changes were described as spatial affinity and confluence in location between the phantom hand and the prosthetic hand when worn.

While not wearing the prosthesis, all participants described the phenomenon of their phantom hand “telescoping” (resting closer to the residual limb as opposed to its correct anatomical position) and remaining immobile. P2 described his phantom hand as clenched, paralyzed in a tight claw, as if “floating” near his shoulder. Yet when putting on the neuromusculoskeletal prosthesis, all participants reported experiencing the phantom hand lengthening to closely meet the position of the prosthetic hand. P2 described this experience as “getting an arm”; his phantom hand relaxed and became animated once again. The topographical synchrony between phantom hand and prosthesis did not only occur in a static position, but also in motion, participants reported. As P1 articulated, “when I open my (phantom) hand, the prosthesis opens.” P2 and P3 also described greater ease moving their phantom limbs while wearing the neuromusculoskeletal prosthesis. P3 emphasized that this mobility occurred only when wearing the prosthesis:

“When I didn’t have a prosthesis, I couldn’t move anything in the phantom, so it has come back now that I’ve gotten this prosthesis, that I can move the phantom. And when I take off the prosthesis, I can’t move the phantom so easily.”

P2 explained that this synchrony occurs in only a matter of seconds after putting on the prosthesis. P2 described the differences before and after using the neuromusculoskeletal prosthesis as follows:

“With these implanted electrodes, I steer the hand with the right thought…it has become more active. It follows much faster. Earlier (with socket prosthesis and surface electrodes), the hand was almost where the prosthetic hand was, but it didn’t follow… I opened (the prosthesis) so (the phantom hand) opened, but it went very slowly. (With the neuromusculoskeletal prosthesis) it follows almost exactly.”

P2 added that his phantom hand tracked the movement of his prosthetic hand even without not looking at it:

“Even if I sit and hold it out like this, away from the eyes… (the phantom hand) follows.”

The animation of his phantom limb and positional synchrony between phantom and prosthesis contributed to the sense that the prosthetic was part of his body, in P2’s words:

“Now with these implanted electrodes that you control with … thought, I think this also made it feel more like a part of the body, because my phantom hand has become more alive. It follows along in the movements much more similarly to the prosthesis.”

#### Participants Describe Difficulty Distinguishing Between Artificially Elicited Sensory Feedback and Naturally Occurring Phantom Limb Sensation at Times

All participants reported experiencing naturally occurring phantom limb sensations, which they did not categorize as painful, and which sometimes proved challenging to differentiate from the somatosensory percepts elicited via neural stimulation. P2 emphasized that in addition to the artificially elicited sensations, “the phantom hand is there the whole time, and it sends signals too.” P2 and P3 described difficulty distinguishing between the artificial and biological phantom sensations. As P2 described:

“(the phantom hand) vibrates and pulsates, and then to distinguish the sensory feedback (by neurostimulation) from the noise that is in the phantom hand, that’s sometimes difficult.”

In the lab, during neurostimulation tests, P2 experienced challenges distinguishing between the two:

“when one … does the tests, sometimes it’s like ‘okay, do I feel the sensory feedback or was that my phantom hand that just did something?”’

P2 described his phantom limb sensation as “(like) it has slept…like when you’ve sat on your hand and made it go numb.”

P3 described a sensory convergence between the sensation prompted by neurostimulation and the sensation he naturally had in his phantom hand:

“I have had phantom sensation but now all of a sudden, (with the sensorized prosthesis) I pick up something…so…the body, or the brain, understands the connection that when I touch something or hold it, then I feel it in the phantom hand. Now it’s a little harder to know, is it a phantom sensation or an artificial sensation? Is the sensation made by the machine, or is it my brain?”

P3 emphasized the increasing challenge of describing the artificial sensation with language, as well as discerning it from the phantom sensation.

### Category 6: Self-Esteem, Self-Image, and Incorporation of the Prosthesis Into Body

#### Participants Experienced Improvements in Self-Esteem and Self-Image

All participants credited increased time of use, diversity of tasks performed, and improved functionality to the neuromusculoskeletal prosthesis. Along with these improvements, they cited peripheral social and emotional benefits, which in turn yielded shifts in their relationship with their prosthesis. They described these shifts with regards to their body and their identity, in the areas of self-esteem and self-image.

Twelve years of living without a prosthesis, P3 described, led to varying struggles with self-esteem:

“I had some days that were good and other days that were not so good, with my self-image. I almost never wore just a t-shirt, instead it was just something to hide. I had a hard time at the beach. Sometimes it went well, other days you feel like ‘no, let me be.’ And … I was treated differently by people when they saw ‘he only has one arm.”’

Since living with the neuromusculoskeletal prosthesis, P3 remarked, “My self-image has gotten better.” In turn, so has his mood, which he described as at a higher, more sustained level.

P2 noticed a similar improvement in his self-esteem, despite his commitment to accepting his body and “not caring” about “look(ing) different” post-amputation. When probed deeper about what “self-esteem” and “caring” meant to him, he replied:

“(the neuromusculoskeletal prosthesis) means something for self-esteem. If I … investigate myself a bit more, it means a lot more than I want to admit. I want to appear like a person who doesn’t really care about it, but I do probably (care), because it’s tough if (the prosthesis) doesn’t work. It means more than I admit.”

P2 commented upon a shift in how he relates to having two- versus one- arms:

“It’s really strange…but now (the prosthesis) feels like it’s more a natural part of my body, and so it feels stranger to be without it…I am not longer even comfortable without it. I wouldn’t say that I am ashamed to go without an arm, but it is a little harder now than it was then, strangely enough.”

Similarly, P1 grew so habituated to wearing the neuromusculoskeletal prosthesis over the last 6 years that he most noticed its significance to his self-esteem and identity when he removed it:

“I always have (the neuromusculoskeletal prosthesis) on me and when I wear it then I feel like… I have two arms and then it’s more like ‘here I am.’ But take the arm away, then it’s like…as if…this isn’t (participant says his own name).”

P3, reflecting on his experience with limb loss in light of receiving the neuromusculoskeletal prosthesis, described:

“I wouldn’t want to change (what happened). I want to be what I am. There are many who are amputated or have been with other things who want them undone, want to have back how it was earlier, but I don’t want that. And it’s clear, a part of all this is of course also that I have gotten such a functioning… a good prosthesis. I think that does a great deal for self-esteem.”

#### Participants Described Feeling Less “Handicapped”

All participants invoked the word “handicapped” (an unprompted word not used by the interviewer) when asked about any changes in self-identity and self-perception since using the neuromusculoskeletal prosthesis. P1 described the experience as “so good, I don’t feel handicapped.” P1 recalled that, for example, when traveling with a socket prosthesis, he would often remove it because it was cumbersome, sweaty, and uncomfortable. When probed as to what the term “handicapped” meant to him, P1 explained:

“If I have a prosthesis…that works, that is easy to wear, easy to use, then I use it and then I don’t feel so handicapped. Handicapped means that you have a functional reduction that prevents you from doing all the chores, work. I have lost a part of my body. So in that way I am handicapped, but I don’t feel like I am handicapped when I wear this (neuromusculoskeletal) arm. Because I can do many things.”

P2 and P3 echoed P1’s commentary on feeling “handicapped” by the socket prostheses, contrasted by a sense of greater functionality, self-sufficiency, and integration with the neuromusculoskeletal prosthesis. As P2 explained, “The earlier socket prosthesis, it was an aid that I carried. This prosthesis, I don’t carry it; it *is* me. I *have* it.”

#### Participants Considered the Neuromusculoskeletal Prosthesis as Part of Their Body, but Not Always as Part of Their *Self*, and Sometimes as a Tool — Depending on Context

During the interviews, participants were asked to describe their neuromusculoskeletal prostheses in relation to their body and self, using a metaphor or analogy. The question was left purposefully vague as to not lead participants or feed them language.

P2 first described the socket prosthesis as a tool, and then explained the difference with the neuromusculoskeletal prosthesis:

“The earlier socket prosthesis, it was an aid that I carried. This prosthesis (neuromusculoskeletal), I don’t carry it; it *is* me. I *have* it. For me it’s as natural as having glasses. The socket prosthesis, that was more of a tool.”

When asked to clarify whether he considered the neuromusculoskeletal prosthesis a part of his body, P2 responded:

“A little bit. The neuromusculoskeletal prosthesis is not biological, no, but you don’t have to think about it. Socket prosthesis, I had to go and think ‘now I must arrange this so that it fits. Change the strap…’ This (neuromusculoskeletal prosthesis) you put it on, and you don’t do anything more.”

When asked whether he identified the neuromusculoskeletal prosthesis as part of his *self* (identity), P2 was more hesitant:

“Maybe not that far, but along those lines. And now with these implanted electrodes that you control with the right thought, I think this also made it feel more like a part of the body, because my phantom hand has become more alive. It follows along in the movements much more similarly to the movements of the prosthesis.”

P3 described a sense of *ownership* over his prosthetic arm, akin to his own arm:

“My prosthesis is a part of my body…It’s my arm now. The (surface electrode) prosthesis…was like a foreign object. I was almost surprised every time I saw it. But this one is, it’s *my* hand, it is *my* arm.”

The interviewer probed this concept of ownership, asking if the fact that the arm is *his* means that it’s a part of *him*, larger than just his body, but extending to a larger sense of self. P3 responded:

“Sometimes when I pick (the prosthesis) up then it becomes another (separate) arm. But the brain has more to do with these electrodes…it becomes more active thinking and using the prosthesis. I (control) the prosthesis with my brain, but then it becomes more like…I want to use this hand as I use the hand. It becomes more of the same (thing). So it (the arm) becomes more of a body part.”

When the interviewer asked the participants whether they considered their neuromusculoskeletal prosthesis as an external tool, the participants pointed out the importance of context. P1 explained that he felt his prosthesis was more of a tool (as opposed to his hand) when, “I’m about to do something quickly, then I realize that this (prosthesis) is not as fast as a (human) hand,” gesturing to the prosthesis’s delayed responsiveness for quick tasks. P3 responded, “Yes, (the neuromusculoskeletal prosthesis) is a tool, but in the way that this is also a tool,” gesturing to his biological hand, waving the fingers. He said the neuromusculoskeletal prosthesis is no more a tool than his biological hand.

#### Challenges With Durability, Mostly From the Terminal Device, Make Participants Feel Less Integrated With Their Prostheses

The neuromusculoskeletal interface increased the use of the commercially available prosthetic hardware (elbow and terminal device), and thus challenged its durability. For all participants, the degree to which they considered the neuromusculoskeletal prosthesis a part of their body depended upon its functionality. All participants reported experiencing occasions of breakdown or malfunction of the prosthetic elbow and terminal device. “I use this (prosthesis) so much that it breaks down regularly,” P1 explained. P1 attributed this breakdown largely to the prosthesis’s material – plastic – and said that he’d rather have a more durable material, such as metal. All participants expressed most problems with the elbow, which P2 said could not withstand heavy loads. P3 reiterated this weakness: “I am stronger than the prosthesis itself. The elbow can break if I take something too heavy, or it gets worn out.”

When breakdowns happened, participants mailed their terminal device for repairs and often used a spare myoelectric hand in the interim. On one occasion, P3 did not have a spare prosthesis and expressed the challenge of sending away the terminal device to an orthopedic engineer for repairs:

“It’s gone for 2–3 weeks. It is really tough to be without the arm, because it has become such a part of me now. I don’t like the prosthesis when it’s broken, or it doesn’t work as it should…then I can get angry at the prosthesis.”

Battery life of the prosthesis is another limitation. P1 voiced a desire for a more durable battery; his current one only lasts about 8–10 h. He always carried a spare battery with him, in his pocket, to change over in order to last him through the day. Overnight, he charged both.

### Category 7: Social Relations

#### Participants Attributed Peripheral Social and Emotional Benefits to Increased Use and Functional Improvements of the Neuromusculoskeletal Prosthesis

In addition to functional changes in daily life, participants articulated improvements in their social and emotional wellbeing since using the neuromusculoskeletal prosthesis. As P3 explained, “there’s a functional side of it all, and … there’s also an emotional side of it all.” Since using the neuromusculoskeletal prosthesis, P3 has noticed he has far fewer “bad days” spent ruminating about his condition and can therefore be more present and engaged with his family.

All participants reported that family members and friends have positively adapted to their neuromusculoskeletal prostheses. P2 noted that his friends responded to his increased capacity to perform movements and partake in shared activities: “they do not offer to help do things for (me…any more). It’s become so natural (for them too).” P1 likewise described feeling more self-sufficient among friends and coworkers: “I don’t need to always ask for help, I can do (things) myself.” He also noted that, with the neuromusculoskeletal prosthesis, acquaintances or strangers didn’t as readily notice his prosthesis or that he was amputated. P3 reported a similar shift among acquaintances and strangers, and furthermore noticed that, with his neuromusculoskeletal prosthesis, he’s grown more comfortable with telling his story and explaining his situation. Whereas living without the prosthesis he sometimes felt beleaguered and bothered by others’ questions of “what happened,” he found it “fun” to explain his new implanted electrodes and “brain-controlled” prosthesis to those interested. “They think everything is very exciting,” he said with a grin.

## Discussion

A thread running through all observed categories and themes is the degree to which participants incorporated the prosthesis into their daily lives, and by extension what effect this incorporation had on how they consider the prosthesis as a part of their body, self, and identity.

### Mechanical Attachment (Osseointegration) and Control (via Implanted Electrodes) Yield Separate, Distinct Benefits for Participants

In the interviews, participants drew two types of comparisons between their experiences with the neuromusculoskeletal prosthesis and prior prostheses: (1) socket-versus-osseointegration mechanical attachment and (2) surface-versus-implanted electrodes. It was thus critical to maintain the integrity of these two categories by disentangling them in our analysis. All participants received osseointegration prior to the surgical implantation of electrodes, ranging from months to years. It should also be noted that participants received the neural sensory feedback for home use (i.e., not confined to the laboratory) relatively recently. These temporal considerations introduce an element of chronology which may or may not have influenced and produced difference among participants’ experiences (i.e., varying degrees of adaptation and familiarity with the neuromusculoskeletal prosthesis’ use and function).

With regard to the mechanical attachment of the prosthesis to body, the benefits of osseointegration have been reported at length, particularly regarding improvements in functionality with resultant increased engagement in life activities (Lundberg et al., 2011; Hansen et al., 2019) as well as the challenges of training and adapting (Hansen et al., 2019). Our participants’ reports of enhanced mobility and improved connection between stump and prosthesis post-osseointegration also indicated a greater sense of comfort and overall bodily balance. Furthermore, in emphasizing the ease with which they were able to remove and put on the osseointegrated prosthesis, participants drew connections between improved mechanical attachment and increased use throughout the day. Consistent with the findings of the only two other known qualitative studies focusing on osseointegrated prostheses and patient experiences (Lundberg et al., 2011; Hansen et al., 2019), our findings suggest an enhanced sense of energy, engagement, and positive affect.

Yet unlike the aforementioned qualitative studies on osseointegration for skeletal attachment (Lundberg et al., 2011; Hansen et al., 2019), neuromusculoskeletal prostheses introduced the additional elements of implanted electrodes for reliable control and intuitive sensory feedback (Ortiz-Catalan et al., 2020). Beyond mechanical attachment, participants commented upon improvements in motor control with implanted electrodes, emphasizing the reduction of electromagnetic interference they experienced with surface electrodes. Most notable was participants’ use of agentive language (i.e., “it’s *me who decides*”) linked to the movement and control of the prosthesis. This sense of agency furthermore engendered a greater degree of trust that the prosthesis would behave according to users’ intentions. Increased trust influenced participants to use their prosthesis in situations where they would not have otherwise with surface electrodes, leading them to take greater risks with their neuromusculoskeletal prosthesis (i.e., carrying fragile objects, like glasses and smart phones). As such, the implanted electrodes seem to have raised both patients’ confidence in and expectations for the degree of control they can expect of their prostheses. Previous studies have suggested that distrust or degrees of caution and risk aversion toward limb prostheses could be due to early adoption or ongoing device development (Graczyk et al., 2019). This was not observed in our participants owing potentially to the reliability and long-term stability of the neuromusculoskeletal prosthetic system when used unsupervised in daily life.

### The Descriptor “Natural” Carries Different Meanings for Participants Depending on Different Contexts

It is important to note that participants used the word “natural” with differing connotations and degrees of enthusiasm, depending on the context, with regard to: (I) reliable and intuitive control, (II) somatosensory feedback via neurostimulation, and (III) the incorporation of the neuromusculoskeletal prosthesis as a body part as opposed to a separate entity.

(I) Participants described the prosthesis as “natural” with regard to reliably and intuitively controlling its function. To them, a “natural” control was experienced when the prosthesis behaved according to their will consistently and in a timely manner.
(II) Participants hesitated to call the quality of sensory feedback “natural,” choosing instead the words “electric” and “numb” as descriptors. In addition, one of the three participants (P3) emphasized that the discrepancy between the *location* of the sensor on the prosthetic thumb and the felt sensation elsewhere on the phantom hand (third finger) created a less-natural feeling, perhaps due to a cognitive or visual dissonance. Location and quality are two different aspects of what could be considered a natural experience. Technological limitations to selectively stimulate different afferent fiber types make it difficult to produce a natural quality (Ortiz-Catalan et al., 2019), although biomimetic approaches have reported to improve it (Valle et al., 2018; George et al., 2019).
(III) Participants also used the word “natural” to describe their incorporation of the neuromusculoskeletal prosthesis into their body (“now [the prosthesis] feels like it’s more a natural part of my body, and so it feels stranger to be without it…I am no longer even comfortable without it.” – P2).

The ambiguity surrounding the use of the word “natural” underscores the importance of identifying what such generalized descriptors *mean* to participants in different contexts. Previous qualitative studies invoking the term “natural” with regard to describing sensory feedback have not differentiated between these multiple contexts and possible nuances in meaning (Graczyk et al., 2019). This suggests the need for ongoing research on the various possible meanings of the term “natural” and the importance of precision when using it in qualitative and quantitative research on artificially elicited sensation. It also demands a degree of epistemological reflexivity, remembering that terms and words themselves carry a weight and history that condition their use and meaning.

Regarding sensory feedback, participants identified limited benefits and expressed a degree of skepticism as to its utility. Participants spoke about the neuromusculoskeletal prosthesis’ intuitive control and function much more positively (evoking words like “trust” and “natural”) than they did the sensory feedback (which they called “not natural”). Participants prioritized the functional benefits of sensory feedback (i.e., improvements in ease of use to perform tasks) as more important than whether or not the sensation itself felt “natural” in its quality. Still, residual reliance on visual feedback to supplement tactile feedback remained for two participants (P2 and P3), perhaps due to perceived weakness of signal strength. Recent work has shown that the selected neuromodulation strategy (frequency modulation proportional to grip force) was far from optimal, and more biologically inspired approaches yield better results (Okorokova et al., 2018; Valle et al., 2018; George et al., 2019; Mastinu et al., 2020). This is because at the point of contact, a critical instant for object manipulation, the elicited sensation was at its weakest, thus requiring certain cognitive effort to be perceived during dynamic tasks in daily life. This issue has now been addressed by neuromodulation strategies that deliver an easily noticeable sensation at contact and release (Mastinu et al., 2020), as provided by fast adaptive afferent fibers in biological touch (Johansson and Flanagan, 2009). Another ongoing improvement is to allow for participants to detect slippage of an object by employing said noticeable discharges in such events.

Participants drew our attention to the challenge of using language to describe a felt sensation. They also used the same words (“asleep, numb”) to describe the sensory feedback as to describe their naturally occurring phantom limb sensation. This underscores an additional challenge that participants faced in discriminating between these two types of sensation. These observations highlight the challenges and limitations of using language to describe, much less measure, interpret, or assess, sensory experience—speaking directly to a larger epistemological debate on the measurement of sensation, particularly pain (Scarry, 1985).

The observed disconnect between participants’ experiences with control and sensory feedback raises the question of whether higher quality control lessens the need or perceived importance of sensory feedback, a question warranting further research. It should be noted that the stimulation paradigm used to provide sensory feedback, to which these results correspond, was in a rather nascent and imperfect form at the time of the interviews; further work on neuromodulation strategies is currently ongoing to improve the utility of somatosensory feedback in subjects with neuromusculoskeletal prostheses (Mastinu et al., 2020). As such, follow-up research with participants is necessary to determine the relevance and utility of sensory feedback when combined with reliable and intuitive control.

### Increased Use of Neuromusculoskeletal Prostheses in Daily Life Yields Improvements in Both Internal (Body Image, Self-Esteem) and External (Social, Relational) Domains

We observed a tight coupling between participants’ use of the neuromusculoskeletal prosthesis in daily life (category 4) and their self-esteem, self-image, and incorporation of the prosthesis into the body (category 5), both yielding peripheral social and emotional benefits (category 7). Our findings resonate with those of Lundberg and colleagues’ study in that participants reported not only functional improvements, but also existential benefits in perceived quality of life (Lundberg et al., 2011). Performing more diverse tasks for longer durations and more holistically incorporating the prosthesis into daily life seems to have trickle-down effects with regard to patients’ emotional wellbeing and the quality of their social lives. These include a greater sense of involvement in family life and an improved sense of self-sufficiency in tasks where they previously required help. Participants attributed these effects largely to improved control over prosthetic function as opposed to socio-affective elements such as touch. Furthermore, positive perception of the technology appeared to increase participants’ positive self-identification with it. We observed a shift among participants from shame or frustration about their prosthesis or being amputated, toward a sense of “fun” and even pride regarding their neuromusculoskeletal prosthesis, particularly when explaining its capabilities to others.

The field of critical disability studies has contributed significantly to interrogating the categories of “handicapped,” “disabled,” and “impaired” while pointing out their profound social, environmental, and linguistic contingency (Ginsburg and Rapp, 2013). With respect to these concerns, it should be noted that these terms were not used by the interviewer, but rather elicited by participants via free association. Still, participants emphasized feeling “not handicapped” while using the neuromusculoskeletal prosthesis. P1’s self-initiated comments about his relationship to and identification with the term “handicapped” also indicate a shift in self-identification: (“Handicapped means that you have a functional reduction… I don’t feel like I am handicapped when I wear this (neuromusculoskeletal) arm”). His words underscored that the feeling of being “handicapped” can be a subjective state related to degree of bodily function, rather than merely to the state of having lost a limb. P3’s words further enforce this notion, gesturing to a broader shift in relation to the experience of having lost his arm: [“There are many who are amputated … who … want to have back how it was earlier, but I don’t …a part of (that) is … I have gotten such a functioning… a good prosthesis. I think that does a great deal for self-esteem”]. Here P3 articulates a link between his functioning prosthesis and this acceptance of, and even a degree of pride in, his post-amputation, prosthetized body.

### “Embodiment” Is Not Static, but Rather Context-Dependent

Participants’ language choices (“part of my body,” “here I am”) raise important questions about proximity of the prosthetic device to their sense of body and self, particularly with regard to embodiment. “Embodiment” is a term used widely in the prosthetics literature, yet often without consensus or precision as to its meaning or definition. We take embodiment to mean not only a sense of ownership over the prosthesis (self-identification with the device as one’s own body), but also a degree of agency over its use (reliable and intuitive control). While participants expressed feeling that their neuromusculoskeletal prosthesis was (at times) part of their body (embodied), they did not necessarily consider it a part of their “self” (a more amorphous category whose distinction from the body remains a long-debated philosophical quandary outside the scope of this article).

Studies in neurostimulation for sensory feedback have reported that participants can experience a sense of ownership of the prosthesis (Schiefer et al., 2016; Page et al., 2018; Rognini et al., 2019). However, it is important to keep in mind that these studies are often acute experiments conducted in controlled laboratory settings, and therefore the effects of ownership (not necessarily embodiment) claimed must also be interpreted as themselves acute and controlled, contained to a specific set of experimental conditions. We must be careful not to extrapolate a sense of ownership and agency (or both) that occur in a cultivated moment or instant to an irreversible, sustained phenomenon. It is for this reason that whereas de Vignemont has defined embodiment as a concomitant sense of ownership and agency, albeit to varying degrees (De Vignemont, 2011), our study suggests an additional element—temporality—must be given greater attention in analyses of embodiment. As people are now, for the first time, living with their neuromusculoskeletal prostheses outside of laboratory contexts and using them freely in their daily lives, embodiment takes on new meaning incorporating context and chronology. The chronic, lived nature of this reality introduces a range of uncontrolled variables, disruptions, and synergies that demand a more nuanced precision of what we mean when we speak about “prosthetic embodiment.”

In our interviews with people living with neuromusculoskeletal prostheses, we found that a sense of embodiment with the prosthesis is conditional and deeply context-dependent, rather than constant or unwavering. For instance, P1, who otherwise refers to his neuromusculoskeletal prosthesis as “my arm,” explained that he realized his neuromusculoskeletal prosthesis was unlike a human hand when he attempted to execute fast movements and found his prosthesis responded more slowly than he intended. Participants’ experiences with mechanical breakdown of their prosthetic hand also underline that breakdown interrupts the sense of incorporating the prosthesis into the body. Frustration and angst (“I don’t like the prosthesis” — P3) can interrupt an otherwise harmonious relationship (“My prosthesis is a part of my body…It’s my arm now”). P3’s language evinces how one’s relationship to a prosthesis is not just a pragmatic one, but also an emotional, affective one. It is in these instances that a disruption occurs in the extent to which an individual identifies with the ownership of, and agency executed over, the device.

Furthermore, our participants did not necessarily seem to distinguish between “tool” and “body” in the dichotomous or mutually exclusive way that has been suggested in other studies of prosthetic embodiment (Murray, 2004; Miller et al., 2014; Gouzien et al., 2017). In using “glasses” as a metaphor for the neuromusculoskeletal prosthesis as an externalized but naturalized essential object, while also saying “it *is* me,” P2 indicates that an object can also be considered part of the body. P3’s somewhat humorous reminder that one’s biological hand can also be considered a tool invites us to more closely examine the assumptions and dichotomies built into the language used to assess embodiment.

### Limitations

Our study is limited by a small (*N* = 3) and homogenous pool of participants with regards to amputation level (transhumeral), gender (male), race (white), nationality (Swedish), and age (mid-40s). In regards to sensory feedback, the participants were provided with a conservative and simplistic neurostimulation strategy (frequency modulation), which has recently been found suboptimal (Mastinu et al., 2020). Taken together, these limitations constrain the generalizability of these findings to other patient populations, genders, amputation level, neural sensory feedback systems, and prosthetic devices.

The three participants, in being the first people implanted with the neuromusculoskeletal system, have received close interaction with experimenters that may differ from the later downstream population of general users. However, this scenario is not unique to our study and is often the case of those who volunteer to participate in early clinical trials and use of highly experimental biotechnologies. Sociologist Everett Rogers’s “diffusion of innovations theory,” first written in 1962, provides a framework to understand the way an innovation is adopted in a social system over time (Rogers, 2003). In this framework, we can understand the three participants as part of the first category of “innovators,” who are often willing to take risks, interested in the technology, and sometimes more positively inclined toward the intervention. While this must be kept in mind in interpretation of the results, we still hold the experiences of these patients as valuable indicators of how people will live with and experience the neuromusculoskeletal prosthesis. Ours is an upstream study in the evolution of this innovation; our findings can be used to guide future design as well as therapeutic and rehabilitative interventions as the technology continues to be adapted for a wider population of users. Furthermore, despite participants’ relative homogeneity and access to clinical service, even among these three users we found differences and nuances in users’ opinions and values based on their lived experiences and contexts. These differences and nuances are noted and, along with shared experiences, form the substance of this study’s analysis.

This is the first account of a long-term implementation of such an integrated neuroprosthetic limb system. These three participants were the first people to permanently utilize implanted electrodes to control and sense with a prosthesis in daily life. Therefore, the importance of this study is in its representation of the firsthand experiences of the first to use such an intimately integrated prosthesis independently. Despite these limitations, this study can still give insight into possible ways humans will integrate and interact with sophisticated prostheses as they proliferate in the future.

The interviews were conducted by one interviewer, holding the style, tone, and focus of the interview consistent. The interviewer was not part of the development team and the interviews were conducted in isolation from other participants or the development team. The participants were at no time dependent on the interviewer for treatment or services. This same interviewer and the co-author were the only two analyzers of the data. Coming from two different disciplines—medical anthropology and biomedical engineering—this provides a complementary view on the experiences of humans as social *and* biological beings, as well as on the technical counterparts that make such an integration of human and machine possible.

## Data Availability Statement

The datasets generated for this study are available on request to the corresponding author.

## Ethics Statement

The studies involving human participants were reviewed and approved by Regional Ethical Committee in Gothenburg, Sweden (Dnr # 1098-17). The patients/participants provided their written informed consent to participate in this study. Written informed consent was obtained from the individual(s) for the publication of any potentially identifiable images or data included in this article.

## Author Contributions

AM and MO-C contributed to the conception and design of the study. AM conducted the interviews and translated the interview transcripts from Swedish to English. AM performed initial analysis and identified themes, which MO-C then cross-validated. AM and MO-C derived descriptive coding system. AM coded and analyzed the interview data. AM wrote the first draft of the manuscript. MO-C wrote sections of the manuscript. AM and MO-C contributed to manuscript revision, read and approved the submitted version.

## Conflict of Interest

MO-C was partially funded by grants in conjunction to Integrum AB, which owns a patent on the e-OPRA technology. No employee of Integrum AB vetted any part of this study. The remaining author declares that the research was conducted in the absence of any commercial or financial relationships that could be construed as a potential conflict of interest.
