# Supplementary material for: Neuromusculoskeletal Arm Prostheses: Personal and Social Implications of Living With an Intimately Integrated Bionic Arm
Source: Front Neurorobot. 2020 Jul 24;14:39. doi: 10.3389/fnbot.2020.00039 (PMC7393241; doi:10.3389/fnbot.2020.00039)
Supplement: Supplementary file 1 [file Data_Sheet_1.PDF]

Interview guide – Qualitative Semi-Structured Interviews with e-OPRA patients

Opening questions

Tell me a bit about yourself – (your age, what you do for work, what you enjoy doing in your free time, etc.)

What is the cause of missing one arm?

When was the amputation?

Are you left- or right-handed?

Was this your dominant hand before the accident/amputation?

How long have you used a prosthesis?

What kind of prosthesis do you currently use?

When was your last osseointegration surgery?

When did you receive sensory feedback?

Do you use multiple prosthetic hands? If so, which? And when do you use each?

How much time during the day do you use the prosthesis?

Do you sleep with the prosthesis on?

Prosthesis use and sensation

How do you experience using your prosthesis?

What does the prosthesis mean to you?

> Describe what it means in different scenarios: At home, at work, with family, with friends, with strangers, your self esteem...

How would you describe your prosthesis? Is there an object or person you would use as an analogy to describe it?

Do you feel your prosthesis is a part of: your body? yourself? Or does it feel more like an external tool?

What is your experience of sensation with your prosthesis?

How would you describe this sensation?

How do you make use of the sensation in the prosthesis?

For what activities do you use the prosthesis?

For what activities do you not use the prosthesis?

What, if anything, do you dislike about the prosthesis?

What hopes or expectations do you have regarding the development of prostheses into the future?

Sensory Feedback

Have you taken part in any experiments about sensory feedback in prostheses?

How did you experience the sensory feedback in the experiments?

Did your experiences of sensation change during the experiments? If so, please describe.

Can you describe how the sensory feedback feels when you currently use your prosthesis?

Would you describe the sensation as “natural”? If not, do you believe it is possible for them to become natural?

What are your thoughts about further developments within the area of sensory feedback?

In your opinion, is sensory feedback important? If not, what is the most important thing for a prosthesis to do?

In what situations would you want sensory feedback in the prosthesis?

(In what way do you think this could influence prostheses use? How should this be accomplished (positioning of fingers, grip force, temperature, touch, contact,...)

Phantom sensation

Do you currently have phantom pain?

Do you experience phantom sensations?

What does it feel like? Can you describe the feeling?

How does the experience of phantom sensation change if you wear your prosthesis?

What does it feel like when you touch your amputation stump?

Follow-up questions:

Can you describe that in more detail? Can you give me an example? Can you develop that further? How do you experience that?...
